# Spatial and Temporal Dynamics of Peste des Petits Ruminants Outbreaks and Their Clinical Impact in Small Ruminants in North Shewa Zone, Ethiopia: Implications for Eradication

**DOI:** 10.1155/tbed/9047158

**Published:** 2025-11-11

**Authors:** Enyiew Alemnew Alamerew, Fasil Aklilu, Thomas Cherenet, Zelalem Yitayew, Derib Aydefruhim, Firdawok Ayele, Anmaw Shite

**Affiliations:** ^1^Livestock Research Directorate, Debre Birhan Agricultural Research Centre, Debre Birhan, Ethiopia; ^2^Department of Veterinary Pathobiology, University of Gondar, Gondar, Ethiopia; ^3^Department of Serology, Animal Health Institute, Sebeta, Ethiopia; ^4^Livestock and Fishery Sector Development Project, Ministry of Agriculture, Addis Ababa, Ethiopia; ^5^Diseases Control and Prevention, Livestock and Fishery Office, Debre Birhan, Ethiopia

**Keywords:** eradication, Ethiopia, outbreaks, peste des petits ruminants, vaccination campaign

## Abstract

Peste des petits ruminants (PPRs) is an economically significant disease affecting small ruminants. Owing to its impact on livestock and rural livelihoods, Ethiopia joined the global PPR eradication program and implemented risk-based vaccination strategies in 2018. However, gaps remain in understanding the geographic distribution, seasonal trends, and impact of this disease. To address these gaps, a repeated cross-sectional study was conducted between September 2018 and August 2024 in the North Shewa Zone to confirm PPR outbreaks via rapid antigen detection methods, specifically the pen-side test and dipstick technique. In addition, a retrospective analysis was performed to assess the spatial and temporal distributions of outbreaks, along with associated morbidity, mortality, and case fatality rates. The results revealed 48 confirmed PPR outbreaks over 6 years, occurring in 15 out of 24 districts, with an average of 8 outbreaks annually and a 0.33 proportion of affected districts, indicating low and localized spread. A total of 192 clinical samples were collected from symptomatic sheep and goats, all of which tested positive for PPRV. In total, 6415 PPR cases were documented, with a morbidity rate of 0.72%, a mortality rate of 0.11% (951 deaths), and a case fatality rate of 14.82%. Kewet had the highest number of outbreaks (7), whereas Mojana-Wedera recorded the highest number of cases (2718). Seasonally, 60% of the outbreaks occurred during the long rainy season, leading to 4472 cases and a fatality rate of 16.79%. Yearly trends showed consistent patterns, with 2023 having the highest number of outbreaks (15) and 2021 having the fewest (5). The year 2024 had the highest number of cases (2706). Despite continued viral circulation, the low morbidity and mortality rates, relative to the high transmissibility and fatality rates of the PPR in naïve populations (infection rates of up to 100% and mortality rates of 10%–90%), reflect significant control progress. While PPR remains endemic in the North Shewa Zone, the reduced incidence and clinical impact indicate measurable advancements toward eradication. Nonetheless, recurrent outbreaks across seasons and districts necessitate sustained, adaptive interventions. The study recommends strengthening surveillance, enhancing postvaccination sero-monitoring, and optimizing vaccine allocation to accelerate eradication.

## 1. Introduction

Peste des petits ruminants (PPRs) is a highly contagious, economically significant, and transboundary viral disease that primarily affects small ruminants such as sheep and goats [1, 2]. Caused by PPRV, a member of the *Morbillivirus* genus, the disease is characterized by clinical signs, including fever, oral lesions, diarrhea, and respiratory distress [1].

PPRs was first reported in 1942 on the Ivory Coast, West Africa. Since then, the disease has spread to many parts of the world and is now endemic throughout Africa and Asia [3]. According to the World Animal Health Information System (WAHIS), PPR has been documented in the Middle East, North, West, South, and East Africa, as well as several parts of Asia [3–6]. The countries that reported PPR outbreaks to the World Organization for Animal Health (WOAH, formerly OIE) from 2012 to 2013 included Algeria, Angola, Comoros, Egypt, Tajikistan, Tunisia, the Republic of the Congo, Kenya, Mali, Uganda, Afghanistan, Bahrain, Bangladesh, Benin, Bhutan, Burkina Faso, Cameroon, the Central African Republic, Chad, Eritrea, Ethiopia, Ghana, Guinea, Guinea-Bissau, India, Iran, Iraq, Israel, Kuwait, Nepal, Nigeria, Saudi Arabia, Sudan, Tanzania, Turkey, and Yemen, indicating widespread endemicity [4–6].

In Ethiopia's neighboring countries, high PPR prevalence rates have been reported, for instance, 42.69% in goat farms in Egypt [7], 27.3% in small ruminants in Uganda [8], 33.48% in Bahrain [9], 4.1% in buffaloes in Indonesia [10], and 5.3% in goats in Somaliland [11]. Within Ethiopia, prevalence rates over the past decade have varied widely, ranging from 2.1% to 75.5% across different regions [12, 13]. Specific studies report 46.53% in the Tigray Region [14], 75.5% in Asossa Zone [13], 12.9% in Bale Zone [15], 60.15% in Afar Region [16], 32.5% in Northwest Ethiopia [17], 10.3% in Dera and Gerar Jarso Districts of Oromia [18], 32.1% in Borena Zone [19], and 60.8% in the Amhara Region [20].

PPRs has profound economic and agricultural implications, particularly in regions like Ethiopia, where livestock are central to rural livelihoods and food security [1, 2, 21]. The disease is highly contagious and leads to severe morbidity and mortality among infected animals [4]. In susceptible populations, the PPR can affect up to 100% of animals, with mortality rates ranging from 10% to 90% [4, 21]. In Ethiopia, the economic impact of PPR is substantial. Field studies have reported flock-level morbidity rates of 83% for sheep and 87% for goats. The mean economic loss per affected sheep flock was ETB 7835 (USD 329 at the 2018 average exchange rate) and ETB 7136 (USD 300) for goat flocks. During outbreaks, losses across all studied flocks were estimated at ETB 319 (USD 13.4) per sheep and ETB 306 (USD 12.9) per goat, with mortality accounting for more than 70% of the total losses. Vaccination costs were estimated at ETB 3 per correctly vaccinated animal [21]. These losses arise from mortality, reduced production, poor-quality animals in live markets, culling, restrictions on transboundary livestock movement, and the costs of vaccination [21–23]. These economic setbacks directly threaten the stability and productivity of rural communities that rely on small ruminant farming for both income and nutrition [1].

Recognizing the scale of this threat, Ethiopia adopted an ambitious goal to eradicate PPR by 2027 through a risk-based vaccination campaign (RBVC) targeting high-risk populations [24], aligning with the global target of eradicating PPR by 2030 [25–27]. Since September 2018, Ethiopia has implemented RBVC strategies focused on regions with high incidence rates. These campaigns use data-driven assessments and rapid antigen detection tools to confirm outbreaks before deploying vaccines [27] to confirm outbreaks before vaccine deployment. The widely recognized live attenuated PPR vaccine [28] was used to immunize 2,917,330 small ruminants, 1,284,339 sheep and 1,632,991 goats during the study period from September 2018 to August 2024 [25].

Despite ongoing control efforts, Ethiopia recorded 554 PPR outbreaks between 2018 and 2022, including persistent cases in the North Shewa Zone of the Amhara Region [19]. These ongoing outbreaks indicate gaps in vaccination coverage, logistical infrastructure, and disease surveillance [25], potentially threatening the 2027 eradication target. Challenges such as limited veterinary services, inadequate funding, security issues, inconsistent vaccine quality, and farmer compliance further complicate disease control [24, 28]. The persistence of PPR in North Shewa underscores the difficulty in achieving full disease control and highlights the need for refined, evidence-based strategies. Additionally, a limited understanding of the geographic spread and seasonal patterns of outbreaks impedes targeted interventions. Moreover, comprehensive data on the morbidity, mortality, and case fatality rates associated with PPR in this region are lacking, which restricts accurate estimation of economic losses and hinders the development of effective policies. Therefore, this study was conducted to provide insight into the spatial and temporal distributions of PPR outbreaks in the North Shewa Zone, Amhara Region, Ethiopia, and to determine the morbidity, mortality, and case fatality rates associated with PPR outbreaks in the study area.

## 2. Materials and Methods

### 2.1. Description of the Study Area

**Figure 1:**
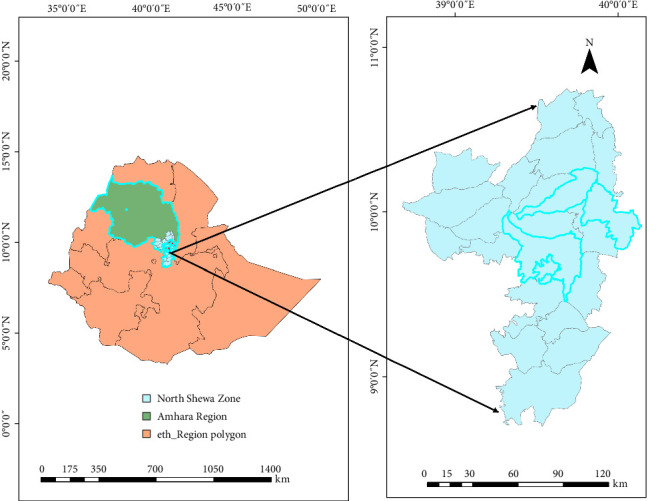
Map of the study areas.

The current study was conducted across all 24 districts of the North Shewa zone, Amhara region, Ethiopia, located at coordinates 9° 46' 8.4ʺN and 39° 40' 4.8ʺE (Figure 1). North Shewa borders several regions: to the south and southeast, it borders the North Shewa and East Shewa zones of the Oromia region, and to the West, North, and Northeast, it borders the East Gojjam zone, South Wollo zone, and the Oromia Liyu zone of the Amhara region, respectively. The eastern boundary of the zone is adjacent to the Afar region (https://latlong.info/ethiopia/amhara-region/north-shewa-zone#north-shewa-zone-latitude-longitude).

Topographically, the zone has elevations ranging from 1500 m in the lowlands to over 3000 m in the highlands. The diverse topography results in varying climates. In highlands, temperatures range between 10 and 20°C, whereas in lowlands, temperatures can reach 20–30°C. The zone experiences bimodal rainfall patterns, with a long rainy season from June to September, a shorter rainy period from February to May, and a dry season from October to January. The average annual rainfall in the zone ranges from 1085 to 1199 mm [29]. Agriculture is the mainstay of the zone economy, with livestock farming, particularly of cattle, goats, and sheep, playing a key role. The zone is marked by mountainous terrain and deep valleys, which support various ecosystems (https://latlong.info/ethiopia/amhara-region/north-shewa-zone#north-shewa-zone-latitude-longitude). North Shewa has a large, small ruminant population of 2,738,402, the second largest in the Amhara region, and boasts the largest sheep population in Ethiopia, contributing significantly to the national total of 90,396,066 sheep (Central Statistical Agency, 2020).

### 2.2. Study Population

The study population consisted of sheep and goats from all districts of the North Shewa Zone, Amhara Region, Ethiopia. The animals were owned by smallholder farmers, as well as governmental and nongovernmental organizations. Smallholder farmers manage their animals under an extensive system, where they graze on fallow lands and crop residues throughout the day, typically without supplementary feed, and are sheltered at night. In contrast, animals owned by governmental and nongovernmental organizations were managed under a semi-intensive system, with supplementary feed provided in addition to grazing.

### 2.3. Study Design

This study employed a repeated cross-sectional design to confirm PPR outbreaks from September 2018 to August 2024. Additionally, a retrospective analysis was conducted using data on PPR outbreaks and case reports during the same period.

### 2.4. Data Collection

#### 2.4.1. Sample Collection and Antigen Detection

A total of 192 samples were collected from 48 confirmed PPR outbreaks, comprising 144 lachrymal and 48 nasal swabs. From each outbreak, four samples were taken, three from the lachrymal and one from the nose, on the basis of a proportional sampling strategy constrained by the cost of rapid antigen detection test kits. Sampling targeted clinically affected small ruminants showing typical signs of PPR, such as oral lesions, diarrhea, ocular and nasal discharge, respiratory distress, and fever above 40°C. Priority was given to animals in the early stage of infection (1–2 days of illness), when watery ocular or nasal discharge coincides with peak antigen detectability. Lachrymal samples were prioritized because of the higher viral load in ocular secretions during early infection and the ease of sample collection under field conditions. Nasal swabs, included once per outbreak, provide supplementary diagnostic coverage, particularly when lachrymal results are inconclusive [26].

Two field tests were employed to improve diagnostic accuracy. As the antigen detection window of PPR is short, beginning approximately 2 days before fever onset and peaking 1–3 days after fever onset, early sampling is essential. Collecting both lachrymal and nasal samples within this window increased the likelihood of identifying active infections. The initial field diagnosis was based on clinical signs, including abortion, dullness, coughing, lip crusting, erosive or necrotic gum lesions, dyspnea, diarrhea, nasal congestion, and mortality. These typically appear 6–12 days post-infection and are accompanied by rising rectal temperature and ocular/nasal discharges [30, 31].

### 2.5. The PPR Rapid Diagnostic Test

Using a sterile swab provided in the kit, the swab was gently rubbed against the conjunctiva inside the third eyelid and lower eyelid, taking care not to touch the eye. The swab was then placed into a plastic vial containing 30 drops of buffer and agitated to mix with the buffer. Rapid antigen detection tests were conducted via the Pirbright Institute Peste-Test UK rapid field test kit: the pen-side test kit technique and the dipstick technique. The laboratory procedure adhered to the manufacturer's guidelines. Pen-side test kit technique: Before the sample was applied, the kit was removed from the packet, placed on a level surface in a shaded area, away from direct sunlight, and allowed to stand for 5 min. The sample was then applied to the test device [26].

**Figure 2:**
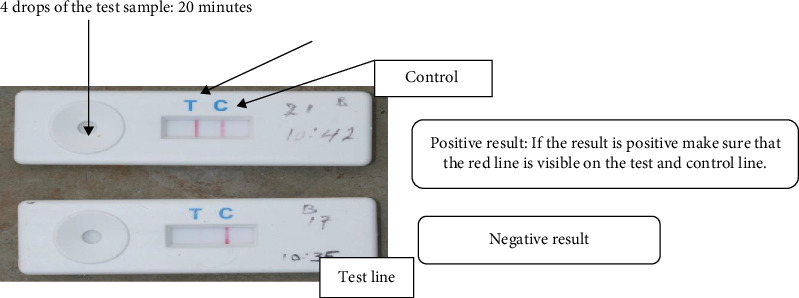
Rapid antigen detection kit procedure.

Dip stick technique: A sample is collected by rubbing the swab against the conjunctiva inside the third eyelid and lower eyelid. Nasal discharge can also be used as an alternative method. Twelve drops of buffer were added to the provided tube. The sampled swab was dipped into the tube and then gently squeezed and shaken to mix it with the buffer. The dipstick was inserted into the mixing tube in the direction of the arrow and left for up to 20 min. After the allotted time, the result is checked. A positive result is indicated by a visible red line on both the test and control lines (Figure 2) [26].

### 2.6. Retrospective Data: PPRs Outbreaks and Vaccination Trends

The data for this study were collected via semistructured questionnaires. This study focused on evaluating the RBVC, gathering information on the starting years of the PPR-RBVC, vaccination trends, the number of small ruminants vaccinated annually, and data on PPR outbreaks, cases, deaths, and the population at risk. These data covered the period from the start of the RBVC to the data collection period (September 2018–August 2024) across all districts in the North Shewa Zone. A 6-year retrospective dataset on PPR outbreaks and vaccination trends was obtained from the North Shewa Zone Animal Health Office through the Disease Outbreaks and Vaccination Activity Reports (DOVAR) system. The data, organized by month, year, and district, include details on PPR outbreaks (cases, deaths, and population at risk) and vaccination figures (number of sheep and goats vaccinated) (Supporting Information 1: Annex S1).

### 2.7. Data Management and Statistical Analysis

The data from field sampling and retrospective data were entered into Microsoft Excel for management, data visualization, and calculation of epidemiological parameters, including the frequency of outbreaks, morbidity, mortality, and case fatality rates of PPR. These parameters were calculated via the following formula: Morbidity rate = (number of cases/total population at risk) × 100. Mortality rate = (number of deaths/total population at risk) × 100. Fatality rate = (number of deaths/number of cases) × 100 [32]. The spatial distribution of the PPR outbreak was created via ArcGIS software version 10.5.

## 3. Results

**Figure 3:**
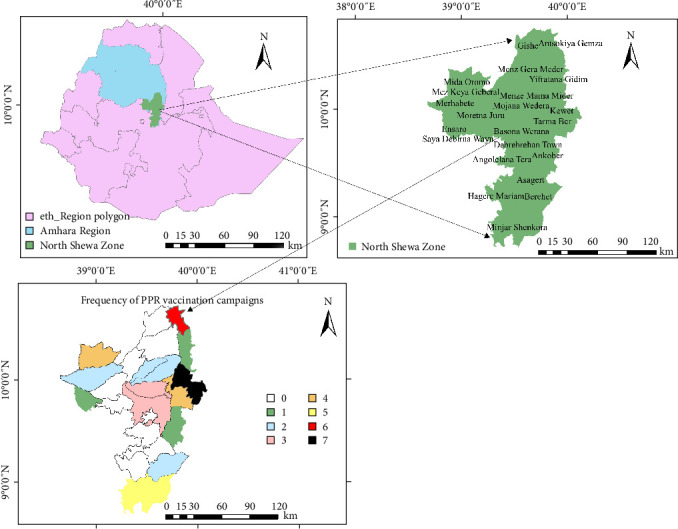
Frequency of PPR outbreak in the study districts from 2018 to 2024.

During the study period, a total of 48 PPR outbreaks were reported in the North Shewa Zone, spanning 15 out of 24 districts (Figure 3). The results indicated that the proportions of disease outbreaks during the study period and across districts were 8 and 0.33, respectively, suggesting a very low occurrence of PPR outbreaks. This reflects the strong commitment and progress of the zone in its efforts to eradicate the disease. In total, 192 samples were collected, including 144 lachrymal and 48 nasal swabs from small ruminants (sheep and goats) exhibiting clinical signs. All the samples were confirmed to be positive for PPRV via the pen-side test and dipstick technique, further confirming the zone's ability to control and eradicate the disease.

Kewet District had the highest frequency, with seven incidents, representing 14.58% of all reported outbreaks. In contrast, nine districts, Debre-birhan, Menz-gishe, Menz-keya, Menz-gera, Angolelana-tera, Asagirt, Hagere-mariam, Siya-wayu, and Moretna-jiru, experienced no reported outbreaks during this timeframe (Figure 3). These outbreaks affected 15 out of 24 districts and resulted in 6415 cases, with an overall morbidity rate of 0.72%, a mortality rate of 0.11% (951 deaths), and a fatality rate of 14.82% (Table 1). This suggests that the impact of the disease was relatively low, as both the morbidity and mortality rates were minimal.

This study highlights disparities in the incidence and outcomes of PPR cases across different districts. The Mojana-Wedera district reported the highest number of cases, with 2718 incidents and 281 deaths. The morbidity rate in this district was 1.15%, with a mortality rate of 0.12% and a fatality rate of 10.34%. The Antsokiya-gemza district followed with 942 cases, exhibiting a morbidity rate of 0.96%, a mortality rate of 0.24%, and a higher fatality rate of 25%. Menz-mama district also showed concerning figures, with 906 cases, a morbidity rate of 6.71%, a mortality rate of 0.27%, and a lower fatality rate of 4%. Notably, this district had the highest morbidity rate (6.71%). The study further revealed variations in the number of deaths and mortality rates. Mojana-Wedera District recorded 218 deaths, whereas Antsokiya-gemza District had 235 fatalities. Ensaro and Menz-lalo District had the highest mortality and fatality rates, at 0.75% and 46.67%, respectively (Table 1). These findings underscore the urgent need for targeted public health interventions in affected districts.

**Figure 4:**
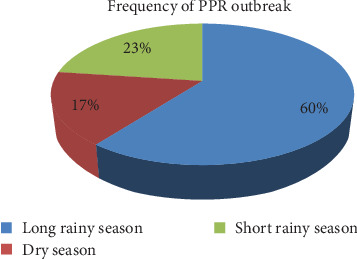
PPR outbreaks by season in the North Shewa Zone.

During the study period, PPR outbreaks were consistently reported across all seasons in the North Shewa Zone. The long rainy season had the highest number of outbreaks, totaling 29 incidents, which represented 60% of all reported cases. The short rainy season was followed by 11 outbreaks, accounting for 23%, whereas the dry season recorded the fewest incidents, with only eight outbreaks, or 17% of the total (Figure 4). Monthly analysis further revealed that outbreaks occurred in nearly every month of the year, with the exception of November. August experienced the highest number of outbreaks, with 13 incidents, representing 27.1% of the total. In contrast, both October and February recorded only one outbreak each, comprising just 4.16% of the total cases (Supporting Information 2: Figure S2). These results highlight a clear seasonal and temporal pattern in PPR outbreak occurrence. This emphasizes the need for targeted disease monitoring, prevention, and control strategies that are season-specific and responsive to the periods of heightened risk.

The long rainy season had the greatest number of cases, totaling 4472, with a morbidity rate of 0.71%, a mortality rate of 0.12%, and a fatality rate of 16.79%. The dry season also presented concerning figures, with 1256 cases, a higher morbidity rate of 2.56%, a mortality rate of 0.19%, and a lower fatality rate of 7.33%. Notably, the dry season presented the highest overall morbidity and mortality rates. Mortality rates varied across seasons, with the long rainy season resulting in the most deaths at 751. The short rainy season was followed by 108 deaths, reflecting a mortality rate of 0.05%. Both the long and short rainy seasons had significant fatality rates of 16.79% and 15.72%, respectively (Table 2). Furthermore, the monthly breakdown of reported PPR cases and associated mortality revealed distinct temporal patterns. August experienced the highest disease burden, with 2857 reported cases and 358 deaths, resulting in a morbidity rate of 0.76% and a mortality rate of 0.1%. Notably, October exhibited the highest monthly mortality rate at 0.43%, while March recorded the highest fatality rate, reaching 39.39% (Supporting Information 3: Table S3). These findings underscore the seasonal and monthly variability in the epidemiological dynamics of PPR and highlight the critical need for time-specific surveillance and targeted intervention strategies to effectively control outbreaks.

**Figure 5:**
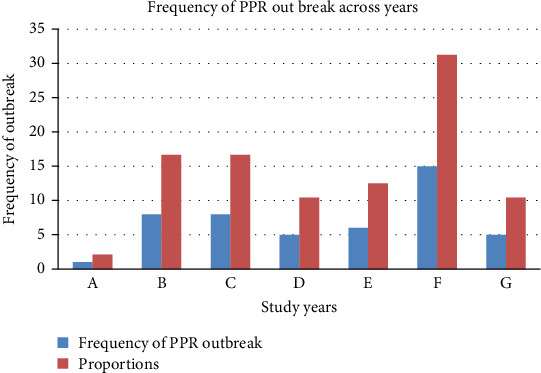
Number of PPR outbreaks by year in the North Shewa Zone. *A* = 2018, *B* = 2019, *C* = 2020, *D* = 2021, *E* = 2022, *F* = 2023, *G* = 2024.

Although the study period included data from 2018 to 2024, data for these 2 years were incomplete. Nonetheless, the results indicate that PPR outbreaks were reported annually in the study areas, with an average of 8.6 outbreaks per year. The highest number of outbreaks occurred in 2023, with 15 cases, accounting for 31.3% of the total outbreaks during the study period. This was followed by 2019 and 2020, each with eight reported outbreaks, together representing 33.4% of the total. In contrast, 2021 recorded the fewest outbreaks, with only five incidents, accounting for 10.4% of the total (Figure 5).

The present study revealed that the highest number of PPR cases occurred in 2024, totaling 2706, with a morbidity rate of 1.61%, a mortality rate of 0.2%, and a fatality rate of 12.16%, although the study was not fully conducted during that year. During the full study period from 2019 to 2023, the lowest number of cases was recorded in 2022, with 166 cases, a morbidity rate of 0.18%, a mortality rate of 0.04%, and a fatality rate of 22.29%. The highest number of deaths also occurred in 2024 (*n* = 329), followed by 2023 (*n* = 300). Notably, 2019 had the highest morbidity and mortality rates, at 1.77% and 0.15%, respectively. The fatality rate peaked in 2023 at 25.55%, followed by 2022, with a recorded rate of 22.29% (Table 3).

## 4. Discussion

Assessing PPR outbreaks is critical to the global effort to eradicate PPRV, as it provides insight into the effectiveness of control measures such as vaccination campaigns and quarantine protocols [33]. In the present study, a total of 48 PPR outbreaks were reported in the North Zone of the Amhara region, Ethiopia, between September 2018 and August 2024, despite the implementation of risk-based vaccination strategies. This highlights the challenges of controlling the disease, even with targeted efforts. A retrospective analysis of national outbreak data revealed that a total of 554 PPR outbreaks were recorded across Ethiopia from 2018 to 2022, including 53 outbreaks in the Borena Zone, Oromia [19]. Furthermore, five outbreaks were documented in the West Arsi Zone from September 2021 to January 2023 [34], and 12 outbreaks were reported across 10 districts in the Southwest Ethiopia Regional State from 2018 to 2022 [35]. These persistent outbreaks underscore the ongoing risks to small ruminant health and their broader implications for food security and economic stability in Ethiopia. These findings emphasize the urgent need for enhanced control measures and coordinated regional and international responses to combat PPR, as continued outbreaks threaten progress toward global eradication.

In the present study, the frequency of PPR outbreaks varied across districts, with higher frequencies observed in the Kewet, Antsokiya-Gemza, Shewa Robit, and Minijar Shenkora districts. These areas may serve as hotspots for PPR due to several factors, including high small ruminant populations, limited access to veterinary services, animal movement patterns, and environmental conditions that facilitate viral transmission. Additionally, their proximity to the Afar Region, where uncontrolled livestock movement for grazing and marketing occurs, likely contributes to the elevated frequency of outbreaks. The high PPR seropositivity rate of 60.15% in small ruminants in Afar further reinforces the link between livestock movement and increased outbreak risk [16]. Uncontrolled livestock movement has long been recognized as a significant factor in the transmission and maintenance of PPRV [22, 23]. Similarly, previous studies have highlighted that regions with dense livestock populations and limited veterinary services are more vulnerable [25, 26]. Similarly, the Horn of Africa, including Kenya and Sudan, also experiences recurrent PPR outbreaks influenced by livestock movement, climatic factors, and insufficient vaccination coverage [29].

These findings align with patterns observed in other high-risk areas of Ethiopia and sub-Saharan Africa, including Southwest Ethiopia and Central Oromia. In these regions, transhumant practices and high livestock density facilitate the transmission of PPR [23, 25]. The variation in outbreak frequency observed in North Shewa mirrors trends observed in neighboring regions, such as Oromia and SNNPR, where districts with high livestock density and limited access to veterinary services tend to experience higher PPR prevalence [20, 21].

Interestingly, the study revealed that nine districts (Figure 3) reported no PPR outbreaks during the study period. This suggests that outbreaks are not uniformly distributed or might be due to subclinical infection and may also be influenced by geographic barriers, herd immunity, local control measures, or environmental conditions that are less favorable for PPR transmission. A previous study noted that areas with high seroprevalence rates, ranging from 85% to 100%, often have lower outbreak frequencies [36]. In contrast, districts with persistent outbreaks, such as Kewet, appear to be more vulnerable despite vaccination efforts, highlighting the complex interplay of vaccination coverage, veterinary infrastructure, and ecological factors.

The retrospective data also enabled us to assess the impact of PPR outbreaks on small ruminants in the study areas, with a focus on morbidity, mortality, and case fatality rates, as well as the role of vaccination campaigns in disease control. The study reported an overall morbidity rate of 0.7%, a mortality rate of 0.1%, and a case fatality rate of 14.8%. These figures are substantially lower than those reported in earlier studies. In naïve populations, the PPR can affect up to 100% of animals, with mortality rates ranging from 10% to 90% [4, 21], highlighting the potential severity of uncontrolled outbreaks. Albina et al. [36] reported morbidity and mortality rates of 16% and 2.7%, respectively, whereas other studies reported morbidity rates as high as 45% [30]. Abubakar et al. [37] subsequently reported morbidity and mortality rates of 30.38% and 15.55%, respectively, and Kardjadj et al. [38] reported rates of 12.2% and 2.5%, respectively. A study in northwest Ethiopia reported morbidity and mortality rates of 51% and 22%, respectively, in sheep and 51% and 25%, respectively, in goats [21]. Similarly, a study in the West Arsi Zone of Ethiopia reported rates of 62.6% morbidity and 19% mortality [34]. In Bangladesh, goats affected by PPR experienced even higher morbidity and mortality rates of 75% and 59%, respectively [39]. Compared with these findings, the present study demonstrated significantly lower morbidity, mortality, and case fatality rates. These findings suggest that ongoing vaccination campaigns and control strategies have played crucial roles in reducing the severity and spread of PPR outbreaks. These lower rates likely reflect improvements in disease surveillance, early detection, and immunization coverage. Overall, the results provide strong evidence that sustained vaccination efforts and effective monitoring systems have mitigated the impact of PPR. This has provided substantial benefits to smallholder farmers in the study zone by protecting their livestock from a disease that can otherwise infect nearly all susceptible animals and cause mortality rates of up to 90% [4].

The overall case fatality rate of 14.83% in this study aligns with previous reports, such as 16.7% [40] and 15.63% [41], but is lower than the 20.3% [38], 100% [30], and 30.3% case fatality rates [34]. Notably, high fatality rates were observed in certain districts of North Shewa, particularly in Menz-lalo (46.7%) and Tarmaber (34.9%), where PPR had a more severe impact. These findings are consistent with those of previous studies in southern Ethiopia, which linked increased fatality to virulent strains of PPR, poor management practices, limited access to veterinary treatment, and inadequate vaccination coverage [35].

The current retrospective data show that PPR outbreaks in small ruminants occur year-round in the study area but are most common during the long rainy season, accounting for 60% of the cases. This seasonal trend may be driven by factors such as livestock aggregation, close confinement, and increased animal movement, all of which create favorable conditions for the spread of the virus [42]. Although outbreaks are often expected during the dry season, due to a lack of adequate pasture and water, long travel distances, and reduced herd immunity [43], this study reveals a different pattern. Notably, the long rainy season coincides with the kidding/lambing period, during which many new, susceptible (naïve) animals are born. By this time, most adult animals are already immunized, either through natural infection or vaccination. As a result, the disease tends to manifest primarily in young animals. Similar patterns have been observed in other parts of Ethiopia, where increased disease incidence during the rainy season has been linked to intensified livestock movement in search of pasture [5, 9, 21, 23, 35, 44].

This seasonal trend is consistent with findings from other regions, including India, where PPR outbreaks also peak during the rainy season due to similar factors such as livestock movement and seasonal conditions [45]. In East Africa, such as Uganda, outbreaks are similarly more common during the wet season, contributing to higher morbidity and mortality [46, 47]. These regional patterns underscore the role of seasonality in the dynamics of PPR transmission across different parts of the world. These seasonal dynamics underscore the importance of seasonally targeted interventions. Vaccination campaigns should be conducted just before high-risk periods, such as the long rainy season, when conditions are more favorable for viral transmission. Additionally, during the dry season, interventions should focus on improving the nutritional status of animals and enhancing veterinary care to mitigate the impact of PPR. Enhanced surveillance and early warning systems that account for seasonal risks should also be prioritized to manage both the direct and indirect impacts of the disease [42].

The current results indicate that PPR outbreaks were reported annually in the North Shewa Zone, with an average of 8.6 outbreaks per year. This finding aligns with previous studies, such as Wendimu et al. [48] in the Central Oromia Region and Fentie et al. [43] in the Amhara region (2010–2014), both of which reported annual PPR outbreaks. The current findings also highlight the yearly variation in the incidence of PPR outbreaks. Specifically, 2023 had the highest number of outbreaks (15 incidents, accounting for 31.3% of the total), followed by 2019 and 2020, each with eight outbreaks (33.4% of the total). In contrast, 2021 had the fewest outbreaks, with only five incidents (10.4%). These findings suggest that while PPR outbreaks remain persistent, their frequency fluctuates annually. The increase in 2023 could be attributed to favorable environmental conditions, such as rainfall or animal movement patterns, which facilitate the spread of the virus. The yearly variation observed in the present study is consistent with trends noted in previous regional studies. For example, Fentie et al. [43] in the Amhara region, Nkamwesiga et al. [49] in Uganda, and Dejene [35] in southern Ethiopia exhibited significant fluctuations, with outbreaks peaking in certain years because of factors such as environmental, epidemiological, and livestock management influences.

Data related to the sex, age, species, and body condition of affected animals are crucial for determining which groups were more severely impacted by PPR and for designing an appropriate control strategy. However, a limitation of the current study is that the data concerning affected animals, such as the number of cases and deaths, were not recorded separately according to sex, age, species, and body condition. This lack of detailed categorization hinders a more precise understanding of the disease's impact on specific animal groups.

## 5. Conclusion and Recommendations

This study, which was conducted in the North Shewa Zone of Ethiopia from 2018 to 2024, offers valuable insights into both the successes and ongoing challenges of PPR eradication efforts. During this period, 48 confirmed outbreaks were reported across 15 districts, affecting over 6400 small ruminants and resulting in nearly 1000 deaths. Although PPR remains endemic in the region, its occurrence has been relatively low and localized, with an average of eight outbreaks per year and a 0.33 annual probability of an outbreak occurring in any given district. Seasonal analysis revealed that the majority of outbreaks occurred during the long rainy season, whereas temporal trends revealed fluctuations in outbreak frequency, peaking in 2023, with the highest number of cases and deaths reported in 2024. Geographically, districts such as Mojana-Wedera and Antsokiya-Gemza bore the highest burden of the disease, whereas Menz-Mama and Ensaro recorded the highest morbidity and fatality rates. These findings underscore the uneven impact of PPR across different locations and seasons. Despite the continued circulation of the virus, the observed morbidity and mortality rates remain relatively low compared with the high rates typically observed in naïve populations, indicating that the vaccination strategy has played a meaningful role in reducing the severity of outbreaks. To further accelerate progress toward PPR eradication, strengthen disease control, support national targets, and contribute to global elimination goals, the following recommendations are proposed:

• Surveillance systems should be strengthened to monitor the spread and timing of PPR via rapid diagnostic tools and efficient data collection methods.
• Postvaccination sero monitoring should be increased to evaluate vaccine efficacy and ensure long-term immunity.
• Community sensitization programs should be launched to raise awareness about the importance of timely vaccination and reporting of clinical signs while addressing vaccine hesitancy and combating misinformation among livestock owners.
• Vaccination and awareness campaigns with seasonal risk patterns should be initiated, particularly before the rainy season, and movement control measures should be implemented during peak outbreak periods to limit disease spread.
• Local veterinary capacity can be built by training and equipping veterinary professionals and animal health workers in outbreak investigations, vaccination, and rapid diagnostics while fostering collaboration among local governments, veterinary services, and pastoralist communities.
• Standardize and digitize outbreak and vaccination data collection for improved accuracy and accessibility, leveraging historical and real-time data to inform policy and optimize resource allocation.

## Figures and Tables

**Table 1:** Spatial distributions of PPR cases, deaths and rates of morbidity, mortality, and fatality in districts.

Study districts	No. of cases	No. of deaths	Population at risk	Morbidity rate (%)	Mortality rate (%)	Fatality rate (%)
Basonawerana	50	10	12,257	0.41	0.08	20
Ankober	16	3	1720	0.93	0.17	18.75
Mojana-wedera	2718	281	237,346	1.15	0.12	10.34
Taramaber	215	75	32,225	0.67	0.23	34.88
Kewet	250	60	294,285	0.09	0.02	24
Shewa-robit	143	33	14,600	0.98	0.23	23.08
Efratana-gidim	62	16	3744	1.66	0.43	25.81
Antsokiya-gemza	942	235	98,365	0.96	0.24	24.95
Menz-lalo	30	14	15,050	0.2	0.09	46.67
Menz-mama	906	36	13,500	6.71	0.27	3.97
Minjar-shenkora	645	118	52,270	1.23	0.23	18.3
Berehet	77	22	72,948	0.11	0.03	28.57
Ensaro	27	5	670	4.03	0.75	18.52
Merhabete	173	15	8750	1.98	0.17	8.67
Mida-weremo	161	28	31,295	0.51	0.09	17.39
Total	6415	951	889,025	0.72	0.11	14.83

**Table 2:** Number of PPR cases and deaths and the rates of morbidity, mortality, and fatality across seasons.

Study seasons	Population at risk	Number of cases	Number of deaths	Morbidity rate (%)	Mortality rate (%)	Case fatality rate (%)
Long rainy season	625,760	4472	751	0.71	0.12	16.79
Dry season	49,039	1256	92	2.56	0.19	7.325
Short rainy season	214,226	687	108	0.32	0.05	15.72
Total	889,025	6415	951	0.72	0.11	14.83

**Table 3:** PPR morbidity rate, mortality rate and fatality rate in the North Shewa Zone across years.

Study years	Populations at risk	Number of cases	Number of deaths	Morbidity rate (%)	Mortality rate (%)	Case fatality rate (%)
2018	13,400	13	2	0.1	0.02	15.39
2019	84,970	1504	123	1.77	0.15	8.18
2020	125,994	565	117	0.45	0.09	20.71
2021	130,024	287	43	0.22	0.03	14.98
2022	92,362	166	37	0.18	0.04	22.29
2023	274,164	1174	300	0.43	0.11	25.55
2024	168,111	2706	329	1.61	0.2	12.16
Total	889,025	6415	951	0.72	0.11	14.83

## Data Availability

The data that support the findings of this study are available from the corresponding author upon reasonable request.

## Supporting Information

Supporting Information 1 Annex S1: This annex includes a questionnaire designed to evaluate the effectiveness of the PPR Risk-Based Vaccination Campaign (RBVC). It covers key aspects, such as the start and strategy of the vaccination campaign and the occurrence of PPR outbreaks. The questionnaire also gathers information on laboratory sample submission for disease confirmation and the outcomes of these efforts.

Supporting Information 2 Figure S2: This figure illustrates the distribution of PPR outbreaks across the study period, showing that outbreaks occurred monthly, except in November. August had the highest number of outbreaks (13 incidents, 27.1% of all cases), whereas October and February each reported only one outbreak (4.16% of total cases).

Supporting Information 3 Table S3: This table provides a detailed analysis of reported PPR cases and mortality rates across months. This highlights that August had the highest number of cases (2857 incidents and 358 deaths), with a morbidity rate of 0.76% and a mortality rate of 0.1%. The table also reveals significant variations in mortality rates, with October having the highest mortality rate (0.43%) and March having the highest fatality rate (39.39%).
