# Supplementary material for: Spatial and Temporal Dynamics of Peste des Petits Ruminants Outbreaks and Their Clinical Impact in Small Ruminants in North Shewa Zone, Ethiopia: Implications for Eradication
Source: Transbound Emerg Dis. 2025 Nov 11;2025:9047158. doi: 10.1155/tbed/9047158 (PMC12626705; doi:10.1155/tbed/9047158)
Supplement: Supporting Information 1 — Annex S1: This annex includes a questionnaire designed to evaluate the effectiveness of the PPR Risk-Based Vaccination Campaign (RBVC). It covers key aspects, such as the start and strategy of the vaccination campaign and the occurrence of PPR outbreaks. The questionnaire also gathers information on laboratory sample submission for disease confirmation and the outcomes of these efforts. [file 9047158.f1.zip › Annex S1.docx]

**Annex S1: Assessing spatial and temporal distribution of PPR outbreaks: Adequacy of the Risk-Based Vaccination Campaign (RBVC)**

1. Did the global PPR eradication program start in the North Shewa zone? A. Yes B. No
2. If yes ( Q1), when was the PPR-RBVC started? ___________
3. Did PPR outbreaks and cases occur? A. Yes B. No
4. If yes (Q3), how many animals were diseased and died in each species per district per year?
5. Did you submit samples for lab confirmation? A. Yes B. No
6. If yes (Q5); what types of samples were submitted A. Serum B. blood, C. postmortem (Kidney, liver), D. swaps (lacrimal and nasal)
7. If yes (Q7); How many samples were submitted, and when and where were they from? ______
8. What types test were applied? __________________
9. If yes (Q6); How many were positive? ____________________
