# Supplementary material for: Spatial and Temporal Dynamics of Peste des Petits Ruminants Outbreaks and Their Clinical Impact in Small Ruminants in North Shewa Zone, Ethiopia: Implications for Eradication
Source: Transbound Emerg Dis. 2025 Nov 11;2025:9047158. doi: 10.1155/tbed/9047158 (PMC12626705; doi:10.1155/tbed/9047158)
Supplement: Supporting Information 1 — Annex S1: This annex includes a questionnaire designed to evaluate the effectiveness of the PPR Risk-Based Vaccination Campaign (RBVC). It covers key aspects, such as the start and strategy of the vaccination campaign and the occurrence of PPR outbreaks. The questionnaire also gathers information on laboratory sample submission for disease confirmation and the outcomes of these efforts. [file 9047158.f1.zip › Annex Sa.docx]

**Annex Sa: Assessing Adequacy of the Risk-Based Vaccination Campaign (RBVC)**

1. Did the global PPR eradication program start in the North Shewa zone? A. Yes B. No
2. If yes (Q1), when was the PPR-RBVC started? ___________
3. What type of vaccination strategy was implemented? __________________________
4. What types of vaccines were used? A. Thermo-stable B Thermo-labile
5. When were you vaccinated? (Months) ____________________________
6. At what interval did you vaccinate the same flock? ___________________________
7. How many districts are covered per year? ______________________
8. How many sheep and goats are vaccinated per year? ________________________
9. Did you check the immune response of the vaccine? A. Yes B. No
10. If yes (Q9), how many animals were sero-positive? ______________________
11. Did you submit samples for lab confirmation? A. Yes B. No
12. If yes (Q11); what types of samples were submitted A. Serum B. Swaps (nasal and lacrimal) C. postmortem (kidney, lung, spleen)
13. 15. If yes (Q12); How many samples were submitted, and when and where were they from? ______
14. What types of test /s has implementing? _______________
15. What is the general approach to vaccination? _______________
