# Supplementary material for: Spatial and Temporal Dynamics of Peste des Petits Ruminants Outbreaks and Their Clinical Impact in Small Ruminants in North Shewa Zone, Ethiopia: Implications for Eradication
Source: Transbound Emerg Dis. 2025 Nov 11;2025:9047158. doi: 10.1155/tbed/9047158 (PMC12626705; doi:10.1155/tbed/9047158)
Supplement: Supporting Information 1 — Annex S1: This annex includes a questionnaire designed to evaluate the effectiveness of the PPR Risk-Based Vaccination Campaign (RBVC). It covers key aspects, such as the start and strategy of the vaccination campaign and the occurrence of PPR outbreaks. The questionnaire also gathers information on laboratory sample submission for disease confirmation and the outcomes of these efforts. [file 9047158.f1.zip › Annex Sb.docx]

**Annex Sb: Assessing Major Constraints on the Progress of PPR Eradication**

This questionnaire aims to assess key constraints affecting the progress of the global PPR eradication program through RBVC at the zonal, district, and peasant association levels. It is designed to identify factors influencing the RBV implementation, examine drivers impacting vaccine coverage, and provide recommendations for corrective actions. If you agree to respond to the following questions, please sign it in the space provided below.

Name of the respondant_____________________________Signiture___________________

1. 1. Is there a national policy for PPR eradication? A. Yes B. No
2. Do you have transparency from national authorities regarding the PPR situation?
   1. A. Yes B. No
3. Do you think that PPR eradication measures are organized and implemented in a coherent way? A. Yes B. No
4. Do you have sufficient national budget allocation for PPR eradication (free vaccine delivery for animal’s owners (cost of vaccine), peridium, others logistic facilities)?
   1. A. Enough B. Limited C. Non-existent
5. Are there national laboratories capable of producing sufficient vaccines to meet the vaccination targets of the National Strategic Plan? A. Yes B. No
6. Do you have adequate vaccine storage capacity? A. Enough B. Limited C. Absent
7. Are there sufficient basic facilities in place for the vaccination chain for PPR control and eradication? I. Vehicles: A. Yes B. Very Limited
   1. II. Cold chain: A. Yes B. Very Limited
   2. III. Other vaccination equipment (syringes, needles, ice packs): A. Yes B. Very Limited
8. Do you have suitable veterinary infrastructure?
   1. I. Sample collection: A. Yes B. No
   2. II. Transport to laboratories: A. Yes B. No
   3. III. Implementation of diagnostic techniques: A. Yes B. No
   4. IV. Data interpretation: A. Yes B. No
9. Are there enough human resources for epidemiological surveillance and vaccination?
   1. I. In numbers: A. Enough B. Moderate C. Limited
   2. II. In skills: A. Enough B. Moderate C. Weak
10. Do farmers' organizations exist? A. Yes B. No
11. How would you rate support from farmers for PPR eradication?
    1. A. Enough B. Weak C. None
12. Have you faced security problems that made field operations difficult or impossible?
    1. A. Yes B. No
13. Are PPR eradication efforts combined with control measures for other small ruminant diseases (provision of therapeutic services for the control of ecto and endo-parasites)? A. Yes B. No
