# Supplementary material for: Spatial and Temporal Dynamics of Peste des Petits Ruminants Outbreaks and Their Clinical Impact in Small Ruminants in North Shewa Zone, Ethiopia: Implications for Eradication
Source: Transbound Emerg Dis. 2025 Nov 11;2025:9047158. doi: 10.1155/tbed/9047158 (PMC12626705; doi:10.1155/tbed/9047158)
Supplement: Supporting Information 2 — Figure S2: This figure illustrates the distribution of PPR outbreaks across the study period, showing that outbreaks occurred monthly, except in November. August had the highest number of outbreaks (13 incidents, 27.1% of all cases), whereas October and February each reported only one outbreak (4.16% of total cases). [file 9047158.f2.docx]

**Figure S2:** **Temporal and Spatial Distribution of PPR Outbreaks across months**

During the study period from January to December, PPR outbreaks occurred every month except November in the North Shewa zone. August had the highest number of outbreaks, totaling 13 incidents, which represented 27.1% of all reported cases. This was followed by July, with 10 incidents accounting for 20.8%. In contrast, October and February each reported only one outbreak, constituting just 4.16% of the total. These findings highlight monthly pronounced variations in outbreak occurrences, emphasizing the need for targeted monitoring and intervention strategies throughout the year (Figure 1).

**Fig. 1:** Frequency of PPR outbreaks by months of the year (2018-2024) in North Shewa Zone
