# Supplementary material for: Spatial and Temporal Dynamics of Peste des Petits Ruminants Outbreaks and Their Clinical Impact in Small Ruminants in North Shewa Zone, Ethiopia: Implications for Eradication
Source: Transbound Emerg Dis. 2025 Nov 11;2025:9047158. doi: 10.1155/tbed/9047158 (PMC12626705; doi:10.1155/tbed/9047158)
Supplement: Supporting Information 3 — Table S3: This table provides a detailed analysis of reported PPR cases and mortality rates across months. This highlights that August had the highest number of cases (2857 incidents and 358 deaths), with a morbidity rate of 0.76% and a mortality rate of 0.1%. The table also reveals significant variations in mortality rates, with October having the highest mortality rate (0.43%) and March having the highest fatality rate (39.39%). [file 9047158.f3.docx]

**Table S3:** Number of PPR cases, deaths and the rate of morbidity, mortality and fatality in during study months

This study provides a thorough analysis of reported cases and mortality rates over several months, revealing key trends in morbidity and mortality. The highest number of reported cases occurred in August, with 2,857 incidents and 358 deaths. The morbidity rate for August was 0.76%, while the mortality rate stood at 0.1%, leading to a fatality rate of 12.53%. Despite the high case count, the low mortality rate indicates that a significant number of cases did not result in death. January followed with 1,134 cases and 63 deaths, with a higher morbidity rate of 4.5% and a mortality rate of 0.25%, resulting in a lower fatality rate of 5.56%. This indicates that while the total number of cases in January was lower than in August, the impact in terms of morbidity was more pronounced. The study also highlights variations in mortality rates across months. August had the highest total deaths (358) with a mortality rate of 0.1% and a fatality rate of 12.5%. July followed with 250 deaths, a mortality rate of 0.16%, and a fatality rate of 25.77%. Notably, October recorded the highest overall mortality rate at 0.43%, while March had the highest fatality rate at 39.39% (Table 1). These findings emphasize the complexities and fluctuations in disease impact over time, underscoring the need for further investigation into the factors influencing these trends and their implications for public health.

**Table-1:** Number of PPR cases, deaths and the rate of morbidity, mortality and fatality in during study months

| **Study Months** | **Populations at Risk** | **Number of Cases** | **Number of Deaths** | **Morbidity Rate (%)** | **Mortality Rate (%)** | **Fatality Rate (%)** |
| --- | --- | --- | --- | --- | --- | --- |
| September | 55,137 | 226 | 64 | 0.41 | 0.12 | 28.32 |
| October | 3,744 | 62 | 16 | 1.66 | 0.43 | 25.81 |
| November | 0 | 0 | 0 | 0 | 0 | 0 |
| December | 20,070 | 60 | 13 | 0.3 | 0.06 | 21.67 |
| January | 25,225 | 1,134 | 63 | 4.5 | 0.25 | 5.556 |
| February | 57,370 | 448 | 36 | 0.78 | 0.06 | 8.036 |
| March | 6,150 | 33 | 13 | 0.54 | 0.21 | 39.39 |
| April | 9,760 | 39 | 10 | 0.4 | 0.1 | 25.64 |
| May | 140,946 | 167 | 49 | 0.12 | 0.03 | 29.34 |
| June | 42,788 | 419 | 79 | 0.98 | 0.18 | 18.85 |
| July | 152,152 | 970 | 250 | 0.64 | 0.16 | 25.77 |
| August | 375,683 | 2,857 | 358 | 0.76 | 0.1 | 12.53 |
| Total | 889,025 | 6,415 | 951 | 0.72 | 0.11 | 14.83 |
